# Supplementary material for: Atomic structures of a bacteriocin targeting Gram-positive bacteria
Source: Nat Commun. 2024 Aug 16;15:7057. doi: 10.1038/s41467-024-51038-w (PMC11329794; doi:10.1038/s41467-024-51038-w)
Supplement: Supplementary file 1 — Supplementary information [file 41467_2024_51038_MOESM1_ESM.pdf]

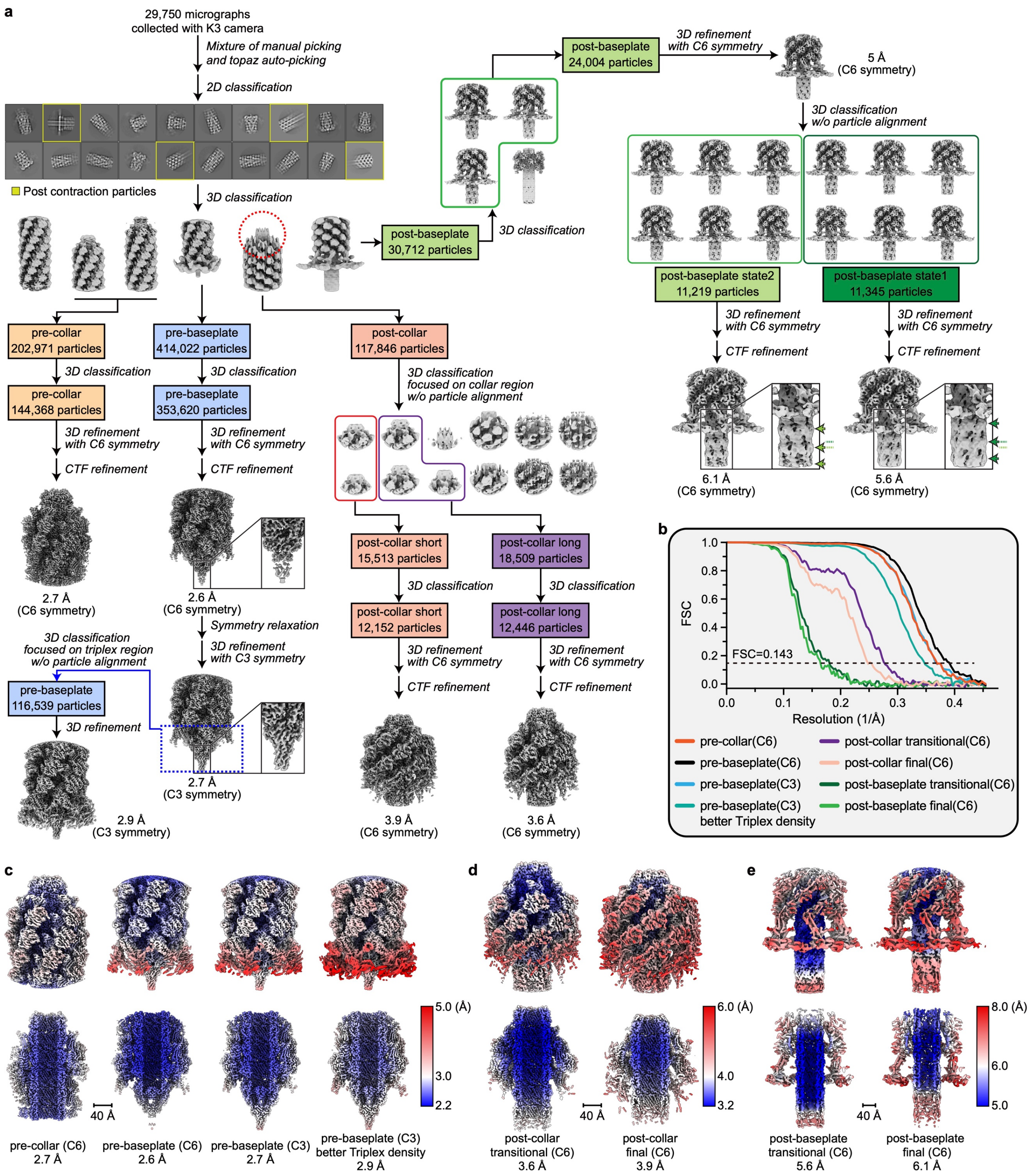

Supplementary Fig. 1 | CryoEM structure determination of the diffocin collar and baseplate in pre- and post-contraction states. a, Data processing workflow (detailed in Methods). **b**, Plot of the Fourier shell correlation (FSC) as a function of the spatial frequency demonstrating the resolutions of final reconstructions. **c**-**e**, Local resolution evaluation of reconstructions of the pre-collar and pre-baseplates (**c**), post-collars (**d**) and post-baseplates (**e**). Post-collar long, post-collar short, post-baseplate state1 and post-baseplate state2 in **a** were defined as post-collar transitional, post-collar final, post-baseplate transitional and post-baseplate final, respectively. See details mentioned in Methods.

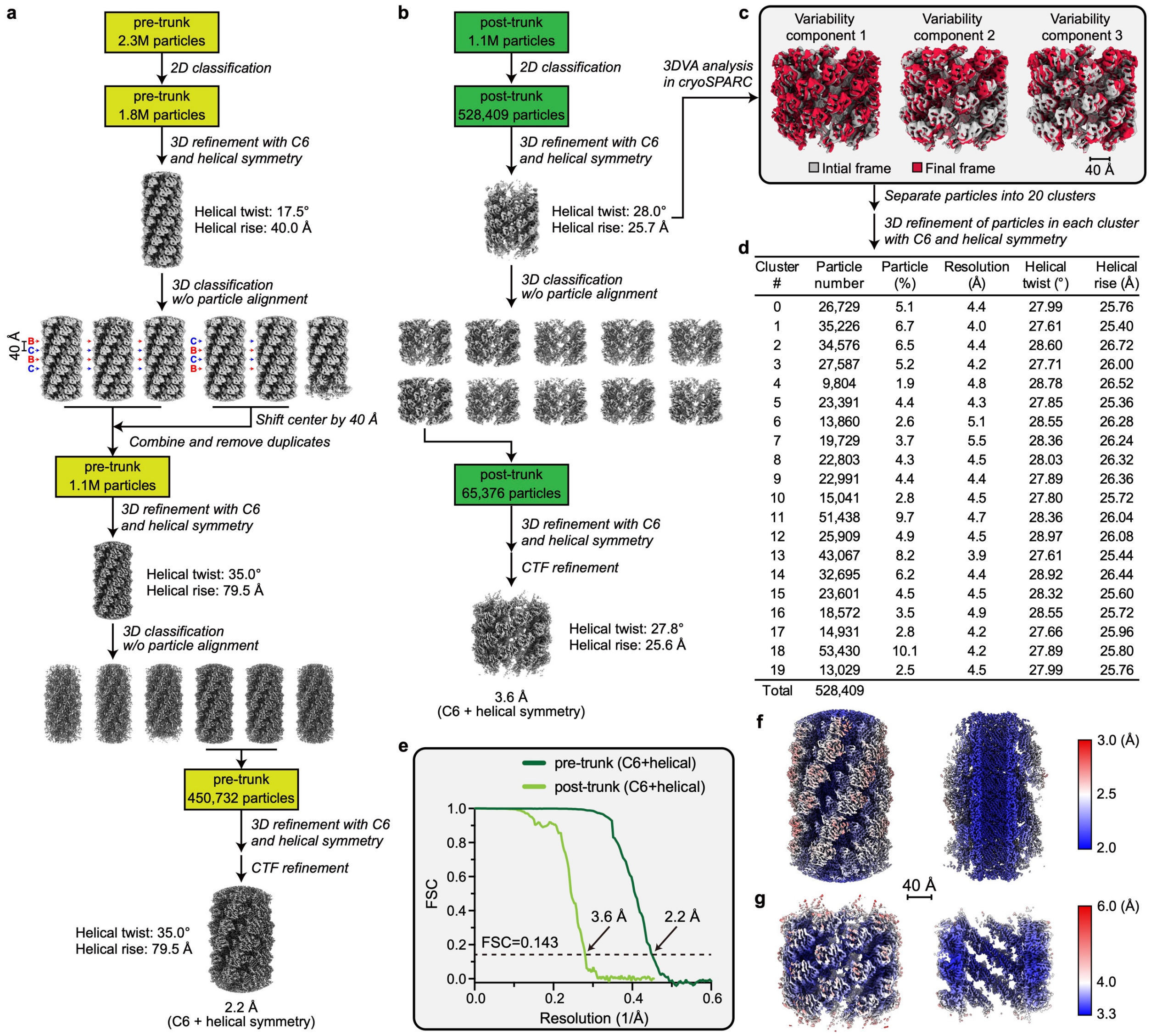

Cluster #	Particle number	Particle (%)	Resolution (Å)	Helical twist (°)	Helical rise (Å)
0	26,729	5.1	4.4	27.99	25.76
1	35,226	6.7	4.0	27.61	25.40
2	34,576	6.5	4.4	28.60	26.72
3	27,587	5.2	4.2	27.71	26.00
4	9,804	1.9	4.8	28.78	26.52
5	23,391	4.4	4.3	27.85	25.36
6	13,860	2.6	5.1	28.55	26.28
7	19,729	3.7	5.5	28.36	26.24
8	22,803	4.3	4.5	28.03	26.32
9	22,991	4.4	4.4	27.89	26.36
10	15,041	2.8	4.5	27.80	25.72
11	51,438	9.7	4.7	28.36	26.04
12	25,909	4.9	4.5	28.97	26.08
13	43,067	8.2	3.9	27.61	25.44
14	32,695	6.2	4.4	28.92	26.44
15	23,601	4.5	4.5	28.32	25.60
16	18,572	3.5	4.9	28.55	25.72
17	14,931	2.8	4.2	27.66	25.96
18	53,430	10.1	4.2	27.89	25.80
19	13,029	2.5	4.5	27.99	25.76
Total	528,409				

Supplementary Fig. 2 | CryoEM structure determination of the diffocin trunk in pre- and post-contraction states and heterogeneity analysis of the post-trunk. a-b, Data processing workflows of the pre-trunk (**a**) and the post-trunk (**b**) (detailed in Methods). **c**, Three-dimensional variability analysis (3DVA) of post-trunk particles reveals contractional movement of sheath layers. Three modes (principal components) of variability were solved. The first and last frames of each mode are shown as gray and red cryoEM density maps, respectively. Videos displaying continuous movement for each mode are available in Supplementary movie 4. **d**, CryoEM reconstructions of 20 clusters of post-trunk particles derived from 3DVA analysis. Particles in each cluster were refined with C6 and helical symmetry to obtain helical parameters including helical twist and rise. **e**, Plot of the Fourier shell correlation (FSC) as a function of the spatial frequency demonstrating the resolutions of final reconstructions. **f-g**, Local resolution evaluation of final reconstructions of the pre-trunk (**f**) and the post-trunk (**g**).

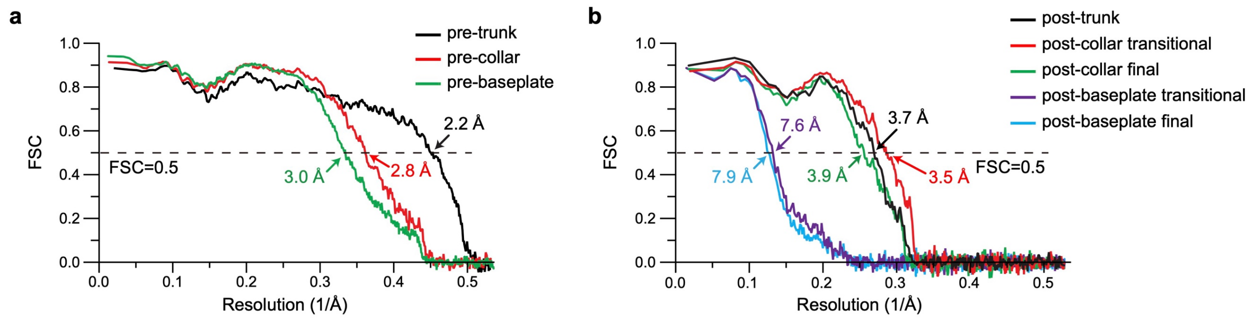

Supplementary Fig. 3 | Evaluation of diffocin models. a-b, FSC coefficients as a function of spatial frequency between models and corresponding cryoEM density maps of the diffocin in the pre- (**a**) and post-contraction states (**b**).

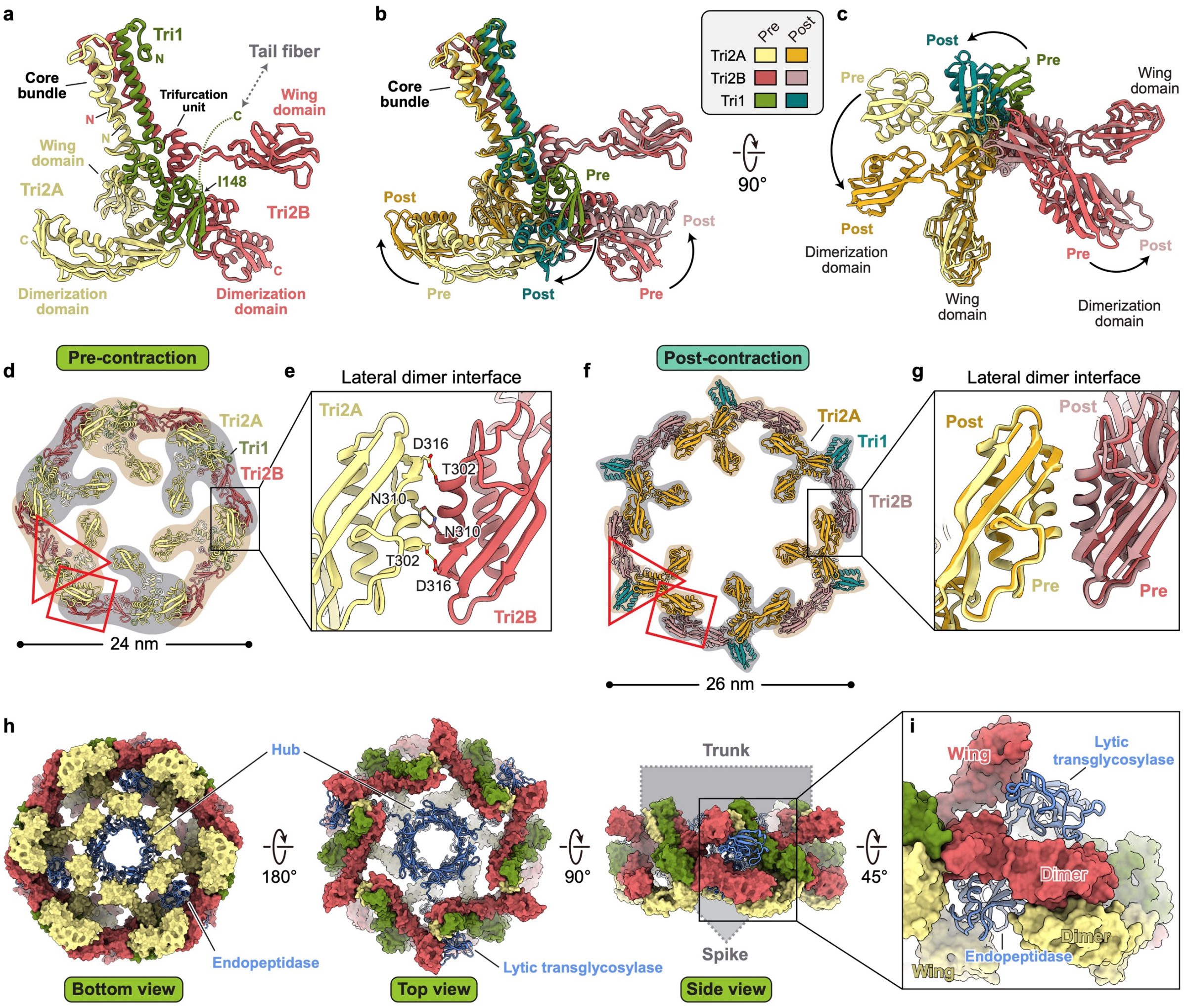

Supplementary Fig. 4 | Structure basis for the expansion of the baseplate wedge during contraction. A, Ribbon diagram of a triplex in the pre-contraction state. A triplex consists of two copies of CD1371 (Tri2A and Tri2B) and one copy of CD1372 (Tri1). The N-terminal regions of three polypeptide chains form the conserved core bundle and trifurcation unit[1], from which the C-terminal wing domains and dimerization domains of Tri2A and Tri2B extrude into different directions. The C-terminal region of Tri1, which connects to the tail fiber, is not resolved in the cryoEM reconstruction. **b**-**c**, Superposition of triplex structures in the pre- and post-contraction states shown in perpendicular views. The two structures were aligned based on the core bundle. **d**, Ribbon diagrams of triplexes forming an iris ring in the pre-contraction state. Adjacent triplexes are distinguished by brown and gray shading. The trifurcation unit and the lateral dimerization interface of triplexes are denoted by a red triangle and a red rectangle, respectively. **e**, Zoom-in view of the lateral dimer formed by the dimerization domains of Tri2A and Tri2B, highlighting key interacting residues. **f**, Ribbon diagrams of an expanded iris ring in the post-contraction state. **g**, Zoom-in view of the lateral dimer in the post-contraction state. The structure of the lateral dimer in the pre-contraction state was superimposed for comparison. **h**, Interactions between the hub-hydrolase (CD1368, shown as ribbons) and the baseplate wedge (shown as surfaces) in the pre-contraction state. **i**, Zoom-in view of the interfaces between the baseplate wedge and two hydrolases of CD1368. The lytic transglycosylase is surrounded by core bundle, lateral dimer and the wing domain of Tri2B. The endopeptidase is surrounded by trifurcation unit, lateral dimer and the wing domain of Tri2A.

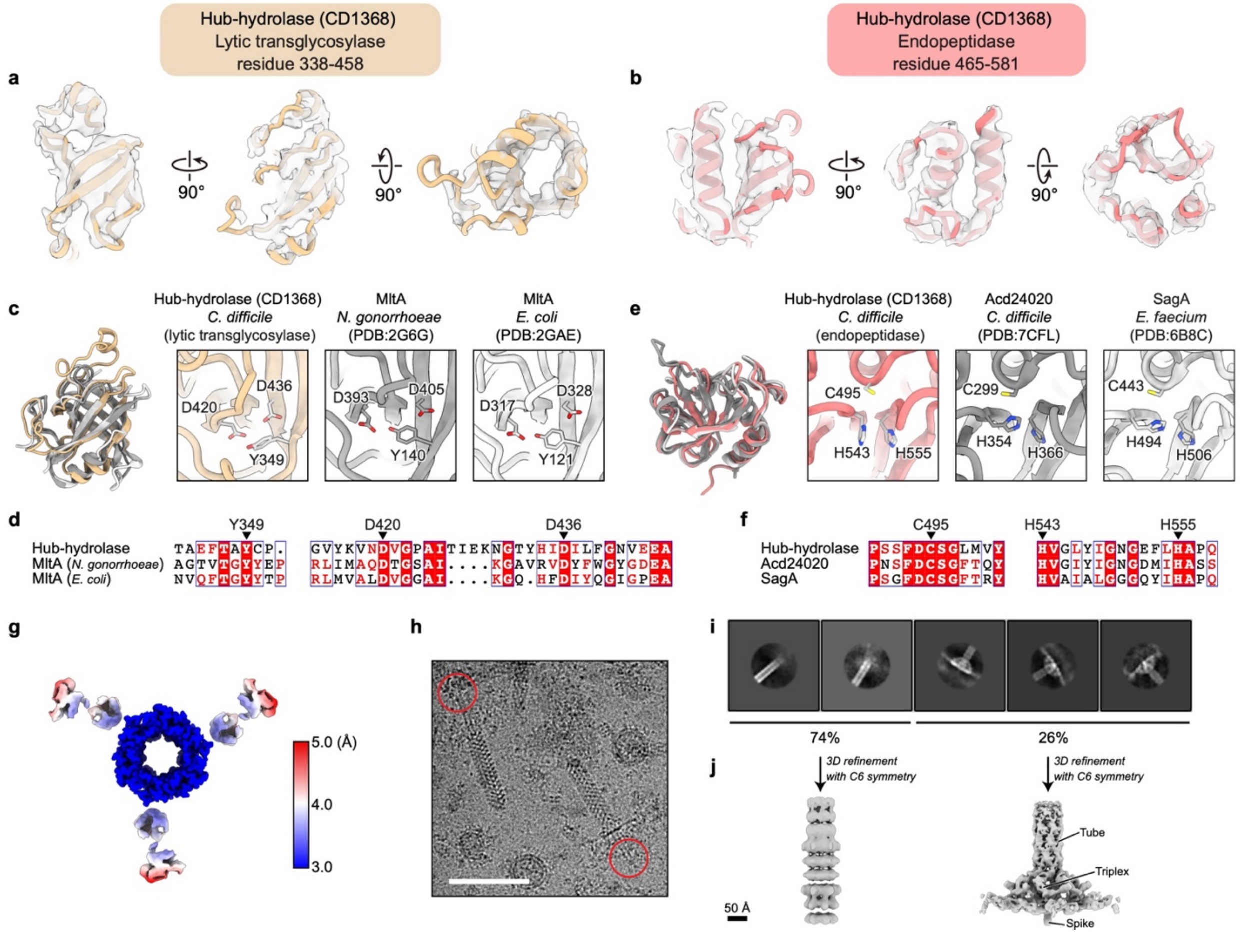

Supplementary Fig. 5 | Structural characterization of the hydrolase domain of the hub-hydrolase (CD1368). a-**b**, Superimposition of cryoEM densities and models of the lytic transglycosylase (**a**) and the endopeptidase (**b**) of the hub-hydrolase. **c**-**d**, Structural comparison (**c**) and sequence alignment (**d**) of the lytic transglycosylase of the hub-hydrolase, MltA from *N. gonorrhoeae* (PDB 2G6G)[2], and MltA from *E. coli* (PDB 2GAE)[2]. Conserved residues of catalytic triad are shown in close-up views in **c** and indicated with black arrows in **d**. **e**-**f**, Structural comparison and sequence alignment (**f**) of the endopeptidase of the hub-hydrolase, Acd24020 from *C. difficile* (PDB 7CFL)[3], and SagA from *E. faecium* (PDB 6B8C)[4]. Conserved residues of catalytic triad are shown in close-up views in **e** and indicated with black arrows in **f**. **g**, Top-view representation of the diffocin hub-hydrolase cryoEM map colored according to the local resolution. Higher resolution areas of the map correspond to blue and lower resolution areas correspond to red. **h**, Representative cryoEM micrograph of the diffocin in the post-contraction state. Scale bar, 100 nm. Spike-proximal tube region subject to further 2D classification (**i**) and 3D reconstruction (**j**) is circled in red. **i**, 2D classification of the post-contraction diffocin spike-proximal region. **j**, 3D reconstructions following C6 symmetry of post-contraction diffocin spike-proximal region. The majority (74%) of particles present as a tube with relatively low resolution due to particle number limitation and without any significant density for the spike or the hub-hydrolase. The minority (26%) of particles present as a tube with intact triplex complexes following contraction.

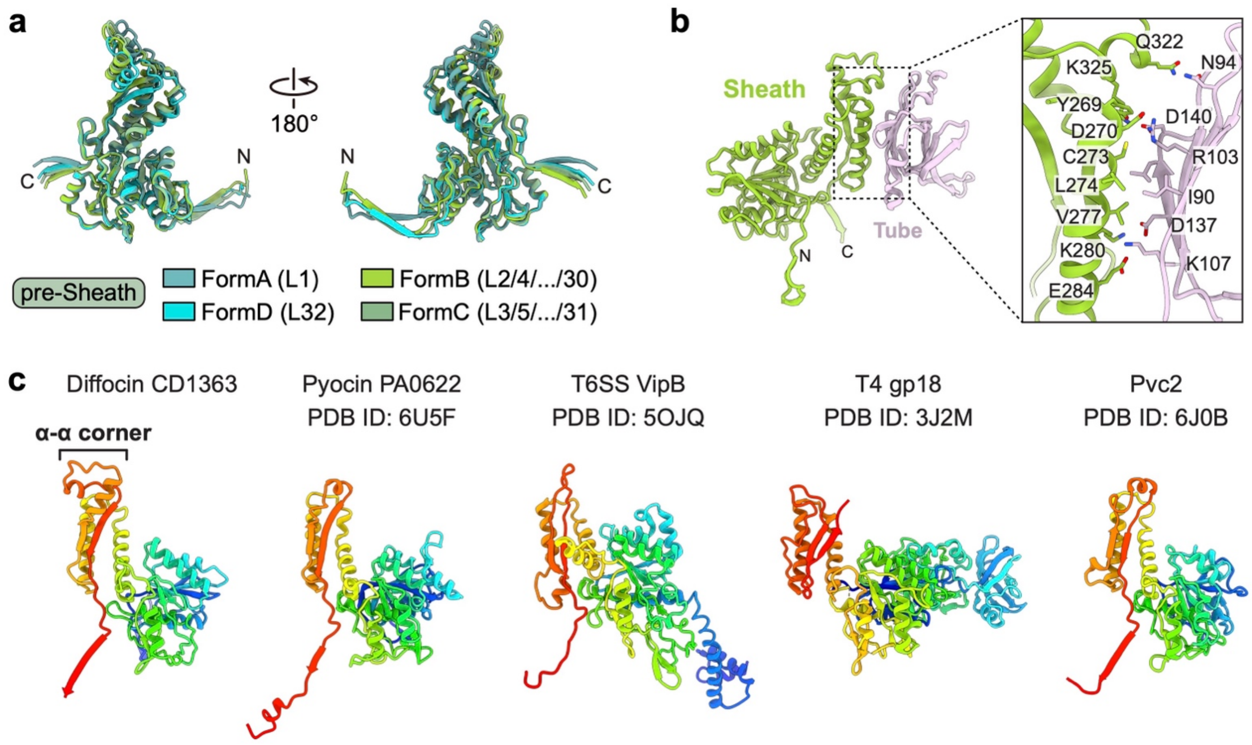

Supplementary Fig. 6 | Structures of the sheath protein. a, Superimposition of four sheath conformers in diffocin structure in the pre-contraction state. $L_{1\text{-}32}$ denotes layers of sheath. **b**, Zoom-in view of the interface between sheath and tube in pre-contraction state. Residues on the interface are labeled and shown in stick representation. **c**, Ribbon diagram comparisons of sheath proteins in diffocin, R-type pyocin[5], T6SS[6], phage T4 [7] and Pvc[8]. The structures are rainbow colored from N-terminus (blue) to C-terminus (red). Only the diffocin sheath presents an α-α corner motif on the tip of the C-terminal β-hairpin.

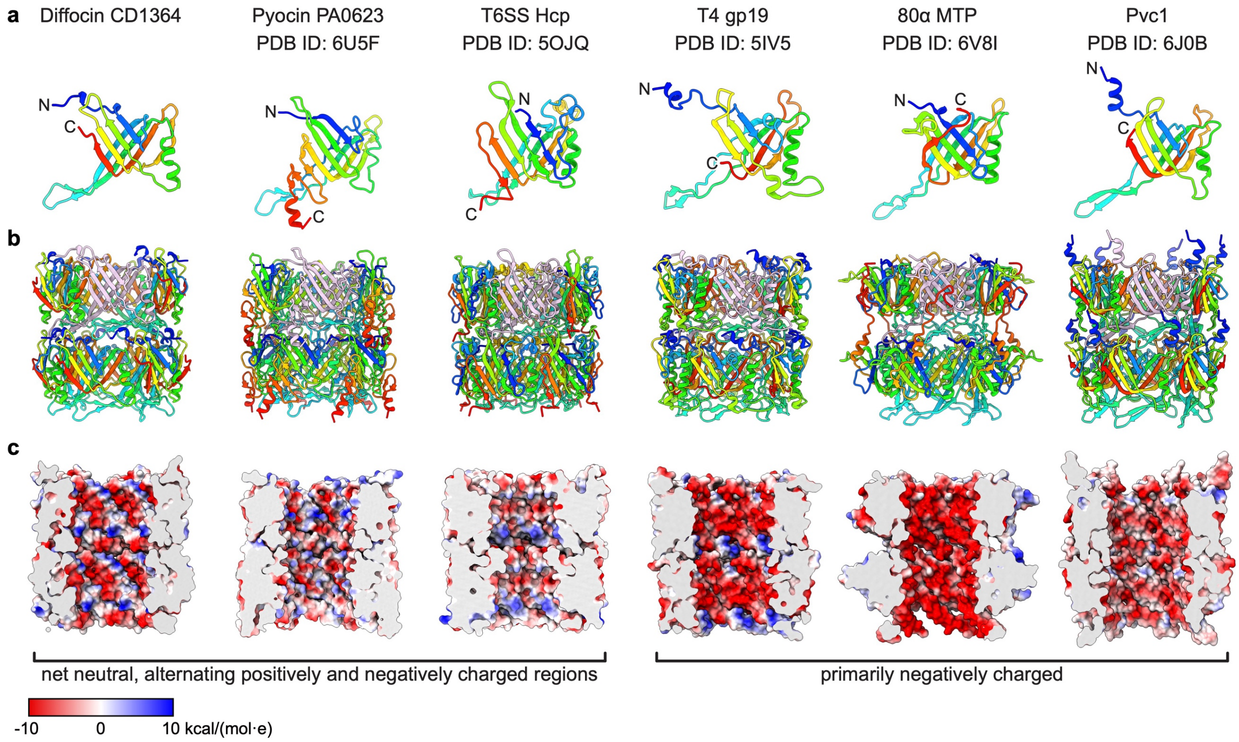

Supplementary Fig. 7 | Structural comparisons of tube proteins and electrostatic diagrams of tube lumen surfaces. a, Ribbon diagrams of tube proteins in diffocin, R-type pyocin[5], T6SS[6], phage T4 [1], phage 80α[9] and Pvc[8]. The structures are rainbow colored from N-terminus (blue) to C-terminus (red). **b**, Ribbon diagrams of two layers of tube proteins. **c**, Electrostatic diagrams of the lumen surface of the tubes. Negative charge is colored in red, positive in blue and neutral in white.

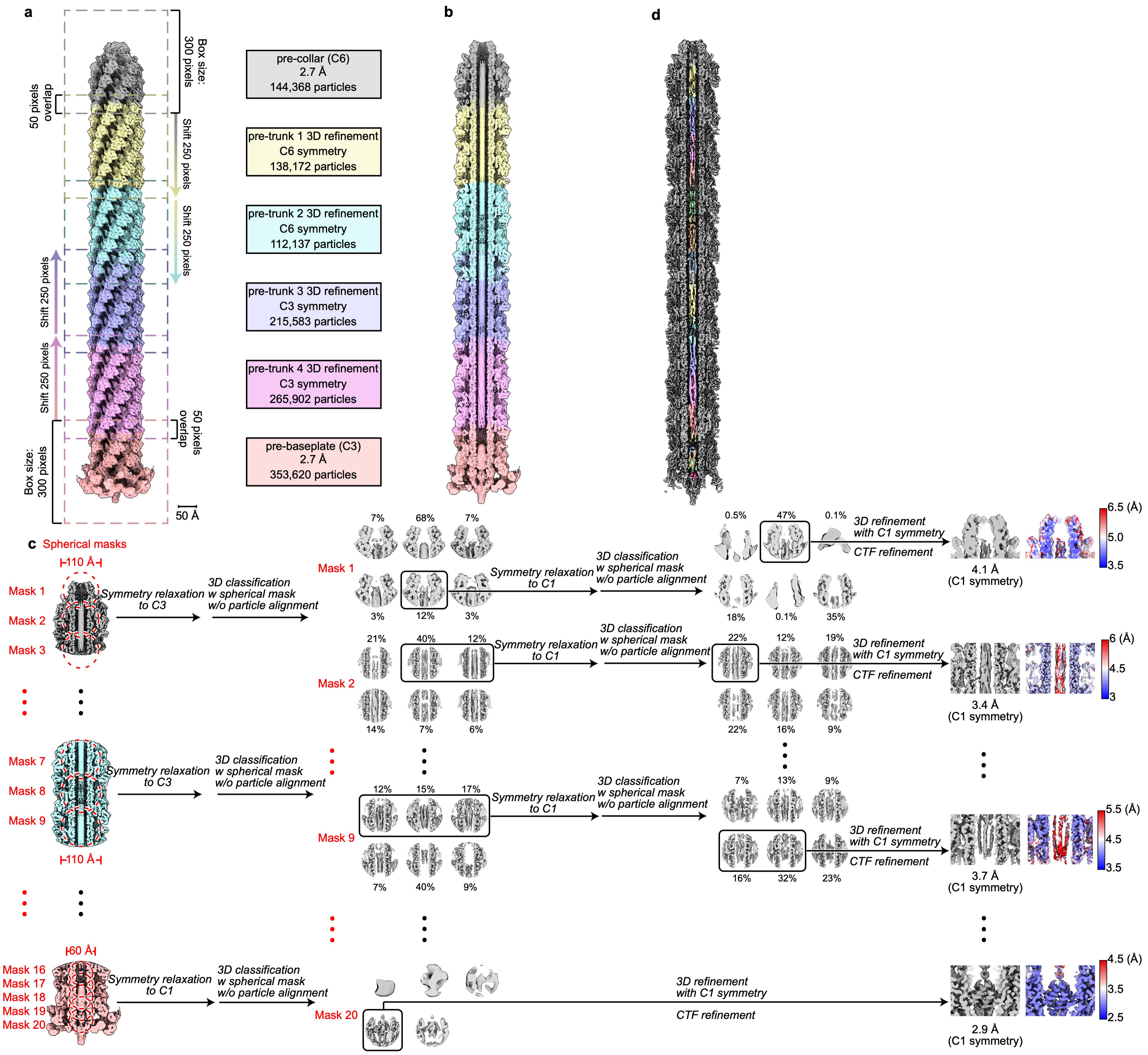

Supplementary Fig. 8 | Data processing workflow of TMP. a, CryoEM density map of the diffocin in the pre-contraction composited by six segments (one collar segment, four trunk segments, and one baseplate segment). Details of the six segments are listed at right side. The density map is colored according to different segments. **b**, Cross-sectional view of composite cryoEM density map of the diffocin presents the diffocin TMP densities before symmetry relaxation and process using featureless spherical masks. **c**, Data processing workflow of the diffocin TMP. See Methods for a more detailed description. **d**, Composite TMP density by twenty density maps (multi-colored) within the cryoEM density of the diffocin (gray).

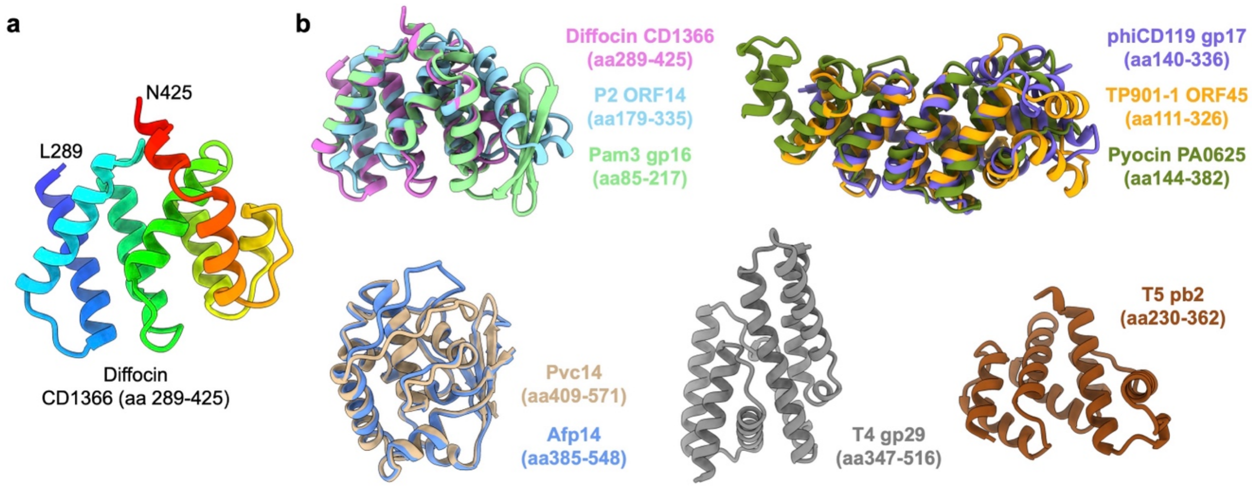

Supplementary Fig. 9 | Globular domains of tape measure proteins (TMPs) predicted by AlphaFold2[10]. a, Ribbon diagram of predicted globular domain of the diffocin TMP. The structures are rainbow colored from N-terminus (blue) to C-terminus (red). **b**, Predicted globular domains of TMPs from phages and phage tail-like nanomachines. Similar structures are overlayed with each other.

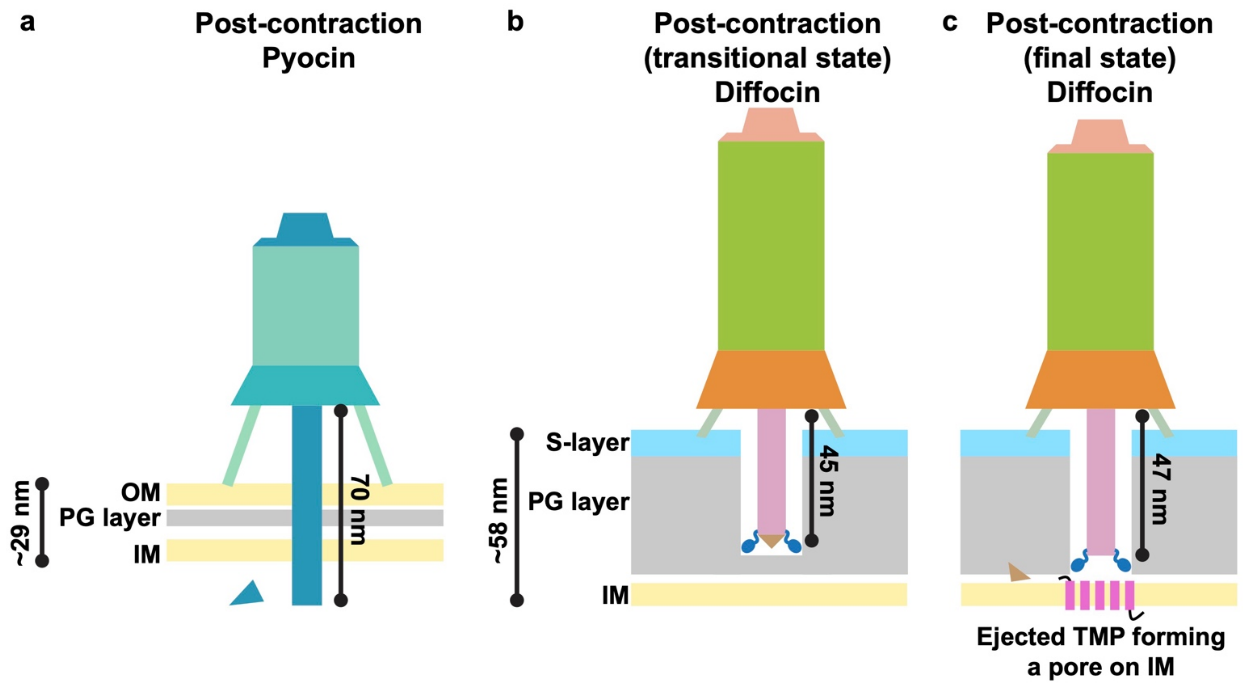

Supplementary Fig. 10 | Comparison of sheath contraction mechanism schematic models of R-type pyocin and the diffocin. a, Model of post-contraction R-type pyocin, targeting Gram-negative *P. aeruginosa*. After the sheath contracts, the spike punctures through the outer membrane (OM), peptidoglycan (PG) layer and inner membrane (IM). **b-c**, Schematic models of the diffocin, targeting Gram-positive *C. difficile*, in the transitional (**b**) and final (**c**) post-contraction states. After the sheath contracts, the spike drills through the S-layer and extends into the PG layer, while simultaneously, the hub-hydrolase degrades the PG layer. A brief pause in the transitional state allows the hub-hydrolase to adequately destroy the PG layer (**b**). The final contraction ejects the TMP from the tube lumen. Five transmembrane helices were predicted on the TMP molecule. A TMP trimer would consequently form a sizeable pore with fifteen transmembrane helices on the IM (**c**). Thus, the overall cell envelope structure of *C. difficile* is compromised, leading to the outflow of ions and disruption of the host cell membrane potential. The thickness of the Gram-positive and Gram-negative cell envelopes and the length of protruding tubes of R-type pyocin and the diffocin are to scale. See Discussion for a more detailed description.

Supplementary Table. 1 | CryoEM data collection, refinement and validation statistics

	Pre-contraction Trunk (EMD-42959) (PDB 8V3X)	Pre-contraction Collar (EMD-42953) (PDB 8V3T)	Pre-contraction Baseplate (C6 sym.) (EMD-42957)	Pre-contraction Baseplate (C3 sym.) (EMD-42958)	Pre-contraction Baseplate (C3 sym. triplex) (EMD-42956) (PDB 8V3W)
**Data collection and processing**					
Magnification	81,000	81,000	81,000	81,000	81,000
Voltage (kV)	300	300	300	300	300
Electron exposure (e–/Å^2)	50	50	50	50	50
Defocus range (μm)	−1.0 – −4.0	−1.0 – −4.0	−1.0 – −4.0	−1.0 – −4.0	−1.0 – −4.0
Pixel size (Å)	0.55*	1.1	1.1	1.1	1.1
Symmetry imposed	C6 + helix	C6	C6	C3	C3
Initial particle images (no.)	2,345,283	202,971	414,022	414,022	414,022
Final particle images (no.)	450,732	144,368	353,620	353,620	116,539
Map resolution (Å)	2.2	2.7	2.6	2.7	2.9
FSC threshold	0.143	0.143	0.143	0.143	0.143
Map resolution range (Å)	2.1 – 2.9	2.5 – 4.0	2.3 – 5.5	2.4 – 6.0	2.5 – 6.0
**Refinement**					
Initial model used (PDB code)	*de novo*	*de novo*			*de novo*, AlphaFold2
Model resolution (Å)	2.2	2.8			3.0
FSC threshold	0.5	0.5			0.5
Map sharpening *B* factor (Å^2)	-70	-70			-70
Model composition					
Non-hydrogen atoms	75,948	76,362			116,790
Protein residues	9,696	9,720			14,709
RNA/DNA Nucleotides					
Ligands					
B factors (Å^2)					
Protein	5.62	69.73			97.53
Ligand					
R.m.s. deviations					
Bond lengths (Å)	0.004	0.008			0.004
Bond angles (°)	0.729	0.731			0.577
Validation					
MolProbity score	1.69	1.77			1.63
Clashscore	5.35	6.7			8.52
Poor rotamers (%)	3.3	4.1			2.6
Ramachandran plot					
Favored (%)	96.82	97.58			97.02
Allowed (%)	3.18	2.42			2.98
Disallowed (%)	0.00	0.00			0.00

	Post-contraction Trunk (EMD-42960) (PDB 8V3Y)	Post-contraction Collar final (EMD-42962) (PDB 8V40)	Post-contraction Collar transitional (EMD-42961) (PDB 8V3Z)	Post-contraction Baseplate final (EMD-42964) (PDB 8V43)	Post-contraction Baseplate transitional (EMD-42963) (PDB 8V41)
**Data collection and processing**					
Magnification	81,000	81,000	81,000	81,000	81,000
Voltage (kV)	300	300	300	300	300
Electron exposure (e–/Å^2)	50	50	50	50	50
Defocus range (μm)	−1.0 – −4.0	−1.0 – −4.0	−1.0 – −4.0	−1.0 – −4.0	−1.0 – −4.0
Pixel size (Å)	1.1	1.1	1.1	1.1	1.1
Symmetry imposed	C6 + helix	C6	C6	C6	C6
Initial particle images (no.)	528,409	117,846	117,846	30,712	30,712
Final particle images (no.)	65,376	12,152	12,446	11,219	11,345
Map resolution (Å)	3.6	3.9	3.6	6.1	5.6
FSC threshold	0.143	0.143	0.143	0.143	0.143
Map resolution range (Å)	3.4 – 5.0	3.4 – 6.7	3.2 – 6.6	4.8 – 9.0	4.5 – 8.5
**Refinement**					
Initial model used (PDB code)	*de novo*	*de novo*	*de novo*	*de novo*	*de novo*
Model resolution (Å)	3.7	3.9	3.5	7.9	7.6
FSC threshold	0.5	0.5	0.5	0.5	0.5
Map sharpening *B* factor (Å^2)	-70	-70	-70	-70	-70
Model composition					
Non-hydrogen atoms	82,290	76,422	76,422	95,334	95,286
Protein residues	10,590	9,732	9,732	12,066	12,060
RNA/DNA Nucleotides					
Ligands					
B factors (Å^2)					
Protein	118.87	102.22	66.44	364.20	331.20
Ligand					
R.m.s. deviations					
Bond lengths (Å)	0.004	0.003	0.003	0.002	0.002
Bond angles (°)	0.748	0.521	0.505	0.473	0.521
Validation					
MolProbity score	1.94	1.61	1.51	1.80	1.99
Clashscore	14.29	8.29	7.08	13.70	18.82
Poor rotamers (%)	3.8	0.2	2.3	0.1	0.3
Ramachandran plot					
Favored (%)	95.98	97.10	97.41	97.15	96.64
Allowed (%)	4.02	2.90	2.59	2.85	3.36
Disallowed (%)	0.00	0.00	0.00	0.00	0.00

* Particles used for the reconstruction were extracted from un-binned micrographs collected in super-resolution mode (pixel size of 0.55 Å).

Reference:

1 Taylor, N. M. *et al.* Structure of the T4 baseplate and its function in triggering sheath contraction. *Nature* **533**, 346-352 (2016). https://doi.org:10.1038/nature17971

2 Powell, A. J., Liu, Z. J., Nicholas, R. A. & Davies, C. Crystal structures of the lytic transglycosylase MltA from N.gonorrhoeae and E.coli: insights into interdomain movements and substrate binding. *J Mol Biol* **359**, 122-136 (2006). https://doi.org:10.1016/j.jmb.2006.03.023

3 Sekiya, H., Tamai, E., Kawasaki, J., Murakami, K. & Kamitori, S. Structural and biochemical characterizations of the novel autolysin Acd24020 from Clostridioides difficile and its full-function catalytic domain as a lytic enzyme. *Mol Microbiol* **115**, 684-698 (2021). https://doi.org:10.1111/mmi.14636

4 Kim, B. *et al.* Enterococcus faecium secreted antigen A generates muropeptides to enhance host immunity and limit bacterial pathogenesis. *Elife* **8** (2019). https://doi.org:10.7554/eLife.45343

5 Ge, P. *et al.* Action of a minimal contractile bactericidal nanomachine. *Nature* **580**, 658-662 (2020). https://doi.org:10.1038/s41586-020-2186-z

6 Wang, J. *et al.* Cryo-EM structure of the extended type VI secretion system sheath-tube complex. *Nat Microbiol* **2**, 1507-1512 (2017). https://doi.org:10.1038/s41564-017-0020-7

7 Fokine, A. *et al.* The molecular architecture of the bacteriophage T4 neck. *J Mol Biol* **425**, 1731-1744 (2013). https://doi.org:10.1016/j.jmb.2013.02.012

8 Jiang, F. *et al.* Cryo-EM Structure and Assembly of an Extracellular Contractile Injection System. *Cell* **177**, 370-383 e315 (2019). https://doi.org:10.1016/j.cell.2019.02.020

9 Kizziah, J. L., Manning, K. A., Dearborn, A. D. & Dokland, T. Structure of the host cell recognition and penetration machinery of a Staphylococcus aureus bacteriophage. *PLoS Pathog* **16**, e1008314 (2020). https://doi.org:10.1371/journal.ppat.1008314

10 Jumper, J. *et al.* Highly accurate protein structure prediction with AlphaFold. *Nature* **596**, 583-589 (2021). https://doi.org:10.1038/s41586-021-03819-2
