# Supplementary material for: Atomic structures of a bacteriocin targeting Gram-positive bacteria
Source: Nat Commun. 2024 Aug 16;15:7057. doi: 10.1038/s41467-024-51038-w (PMC11329794; doi:10.1038/s41467-024-51038-w)
Supplement: Supplementary file 2 — Reporting Summary [file 41467_2024_51038_MOESM2_ESM.pdf]

nature portfolio

Corresponding author(s): Z. Hong Zhou, Jeff F. Miller

Last updated by author(s): Jul 18, 2024

Reporting Summary

Nature Portfolio wishes to improve the reproducibility of the work that we publish. This form provides structure for consistency and transparency in reporting. For further information on Nature Portfolio policies, see our Editorial Policies and the Editorial Policy Checklist.

Statistics

For all statistical analyses, confirm that the following items are present in the figure legend, table legend, main text, or Methods section.

n/a	Confirmed	
☐	☒	The exact sample size (*n*) for each experimental group/condition, given as a discrete number and unit of measurement
☒	☐	A statement on whether measurements were taken from distinct samples or whether the same sample was measured repeatedly
☐	☒	The statistical test(s) used AND whether they are one- or two-sided<br>*Only common tests should be described solely by name; describe more complex techniques in the Methods section.*
☒	☐	A description of all covariates tested
☒	☐	A description of any assumptions or corrections, such as tests of normality and adjustment for multiple comparisons
☐	☒	A full description of the statistical parameters including central tendency (e.g. means) or other basic estimates (e.g. regression coefficient) AND variation (e.g. standard deviation) or associated estimates of uncertainty (e.g. confidence intervals)
☐	☒	For null hypothesis testing, the test statistic (e.g. *F*, *t*, *r*) with confidence intervals, effect sizes, degrees of freedom and *P* value noted<br>*Give P values as exact values whenever suitable.*
☒	☐	For Bayesian analysis, information on the choice of priors and Markov chain Monte Carlo settings
☒	☐	For hierarchical and complex designs, identification of the appropriate level for tests and full reporting of outcomes
☒	☐	Estimates of effect sizes (e.g. Cohen's *d*, Pearson's *r*), indicating how they were calculated

Our web collection on statistics for biologists contains articles on many of the points above.

Software and code

Policy information about availability of computer code

Data collection	Cryo-EM: SerialEM 3.8
Data analysis	Cryo-EM: MotionCor2, CTFFIND4, RELION 4.0, CryoSparc v4.4.1, AlphaFold2, Coot0.8.9.1 , Phenix1.20.1, Chimera 1.17 and ChimeraX 1.6.

For manuscripts utilizing custom algorithms or software that are central to the research but not yet described in published literature, software must be made available to editors and reviewers. We strongly encourage code deposition in a community repository (e.g. GitHub). See the Nature Portfolio guidelines for submitting code & software for further information.

Data

Policy information about availability of data

All manuscripts must include a data availability statement. This statement should provide the following information, where applicable:

- Accession codes, unique identifiers, or web links for publicly available datasets
- A description of any restrictions on data availability
- For clinical datasets or third party data, please ensure that the statement adheres to our policy

The cryoEM density maps and corresponding atomic models have been deposited in the EMDB and PDB, respectively. The accession numbers are listed as follows: pre-contraction collar (PDB: 8V3T [http://doi.org/10.2210/pdb8V3T/pdb] and EMD-42953 [https://www.ebi.ac.uk/pdbe/entry/emdb/EMD-42953]); post-contraction collar in transitional state (PDB: 8V3Z [http://doi.org/10.2210/pdb8V3Z/pdb] and EMD-42961 [https://www.ebi.ac.uk/pdbe/entry/emdb/EMD-42961]); post-contraction collar in final state (PDB: 8V40 [http://doi.org/10.2210/pdb8V40/pdb] and EMD-42962 [https://www.ebi.ac.uk/pdbe/entry/emdb/EMD-42962]); pre-contraction baseplate reconstructed in C6 symmetry (EMD-42957 [https://www.ebi.ac.uk/pdbe/entry/emdb/EMD-42957]); pre-contraction baseplate

reconstructed in C3 symmetry (EMD-42958 [https://www.ebi.ac.uk/pdbe/entry/emdb/EMD-42958]); pre-contraction baseplate focused refinement on triplex region (PDB: 8V3W [http://doi.org/10.2210/pdb8V3W/pdb] and EMD-42956 [https://www.ebi.ac.uk/pdbe/entry/emdb/EMD-42956]); post-contraction baseplate in transitional state (PDB: 8V41 [http://doi.org/10.2210/pdb8V41/pdb] and EMD-42963 [https://www.ebi.ac.uk/pdbe/entry/emdb/EMD-42963]); post-contraction baseplate in final state (PDB: 8V43 [http://doi.org/10.2210/pdb8V43/pdb] and EMD-42964 [https://www.ebi.ac.uk/pdbe/entry/emdb/EMD-42964]); pre-contraction trunk (PDB: 8V3X [http://doi.org/10.2210/pdb8V3X/pdb] and EMD-42959 [https://www.ebi.ac.uk/pdbe/entry/emdb/EMD-42959]); post-contraction trunk (PDB: 8V3Y [http://doi.org/10.2210/pdb8V3Y/pdb] and EMD-42960 [https://www.ebi.ac.uk/pdbe/entry/emdb/EMD-42960]). Source data of the length of diffocin particles are provided with this paper.

Research involving human participants, their data, or biological material

Policy information about studies with human participants or human data. See also policy information about sex, gender (identity/presentation), and sexual orientation and race, ethnicity and racism.

Reporting on sex and gender	N/A
Reporting on race, ethnicity, or other socially relevant groupings	N/A
Population characteristics	N/A
Recruitment	N/A
Ethics oversight	N/A

Note that full information on the approval of the study protocol must also be provided in the manuscript.

Field-specific reporting

Please select the one below that is the best fit for your research. If you are not sure, read the appropriate sections before making your selection.

☒ Life sciences ☐ Behavioural & social sciences ☐ Ecological, evolutionary & environmental sciences

For a reference copy of the document with all sections, see nature.com/documents/nr-reporting-summary-flat.pdf

Life sciences study design

All studies must disclose on these points even when the disclosure is negative.

Sample size	No statistical method was used to predetermine sample size. For cryo-EM study, the sample sizes were determined by densities of particles on cryo-EM grids and the number of collected cryo-EM images. 3D reconstructions of pre-contraction collar, post-contraction collar in transitional state, post-contraction collar in final state, pre-contraction baseplate, post-contraction baseplate in transitional state, post-contraction baseplate in final state, pre-contraction trunk, and post-contraction trunk were calculated from 29,750 images (~4 million particles). The data size is comparable to other studies in this field. The number of particles used for each final map is sufficient to obtain reliable classification and reconstruction results by cryo-EM.
Data exclusions	Cryo-EM particles which have poor qualities or are not interested targets were excluded during 2D and 3D classification. This standard l procedure has been widely used to obtain high resolution cryo-EM structures of biomacromolecules.
Replication	Cryo-EM datasets were collected with multiple samples in separate imaging sessions, yielding consistent results (see Methods section for details).
Randomization	Since this study didn't involve research on living organisms, no confounding factors requiring randomization were expected. No randomization has been performed.
Blinding	Since this study didn't involve research on living organisms, no confounding factors requiring binding were expected. No blinding has been performed.

Reporting for specific materials, systems and methods

We require information from authors about some types of materials, experimental systems and methods used in many studies. Here, indicate whether each material, system or method listed is relevant to your study. If you are not sure if a list item applies to your research, read the appropriate section before selecting a response.

Materials & experimental systems

n/a	Involved in the study	
☒	☐	Antibodies
☒	☐	Eukaryotic cell lines
☒	☐	Palaeontology and archaeology
☒	☐	Animals and other organisms
☒	☐	Clinical data
☒	☐	Dual use research of concern
☒	☐	Plants

Methods

n/a	Involved in the study	
☒	☐	ChIP-seq
☒	☐	Flow cytometry
☒	☐	MRI-based neuroimaging

Plants

Seed stocks	N/A
Novel plant genotypes	N/A
Authentication	N/A
