# Supplementary material for: Atomic structures of a bacteriocin targeting Gram-positive bacteria
Source: Nat Commun. 2024 Aug 16;15:7057. doi: 10.1038/s41467-024-51038-w (PMC11329794; doi:10.1038/s41467-024-51038-w)
Supplement: Supplementary file 4 — Description of Additional Supplementary Files [file 41467_2024_51038_MOESM4_ESM.pdf]

Description of Additional Supplementary Files:

Supplementary movie 1: Overall structure of the pre-contraction diffocin.
A montage structure of the pre-contraction diffocin, highlighting density details of collar, trunk and baseplate regions.

Supplementary movie 2: Computer constellation of atomic models into a pre-contraction diffocin.
Ribbon diagrams of individual subunits are put together to form the model of pre-contraction diffocin.

Supplementary movie 3: Overall structure of the post-contraction diffocin.
A montage structure of the post-contraction diffocin, highlighting density details of the collar at transitional and final states, and the trunk.

Supplementary movie 4: 3DVA analysis of the trunk of post-contraction diffocin revealing conformational heterogeneity.

Supplementary movie 5: Tape measure protein density of the pre-contraction diffocin.
Tape measure protein colored in magenta is present in the sectional view of pre-contraction diffocin. The camera then zooms in and goes through the entire tape measure protein density.
